# Supplementary material for: Multivariate Analysis of the Cotton Seed Ionome Reveals a Shared Genetic Architecture
Source: G3 (Bethesda). 2018 Feb 1;8(4):1147–60. doi: 10.1534/g3.117.300479 (PMC5873906; doi:10.1534/g3.117.300479)
Supplement: Supplementary file 5 [file 1147FileS5.pdf]

Multivariate analysis of the cotton seed ionome reveals a shared genetic architecture

Duke Pauli[*1], Greg Ziegler[†,‡], Min Ren[§], Matthew A. Jenks[**], Douglas J. Hunsaker[††], Min Zhang[§], Ivan Baxter[†,‡], and Michael A. Gore[*]

[*]Plant Breeding and Genetics Section, School of Integrative Plant Science, Cornell University, Ithaca, NY 14853, USA
[†]Donald Danforth Plant Science Center, St. Louis, Missouri, USA
[‡]United States Department of Agriculture–Agricultural Research Service (USDA-ARS), Plant Genetics Research Unit, St. Louis, Missouri, USA
§Department of Statistics, Purdue University, West Lafayette, IN 47907, USA
[**]Division of Plant and Soil Sciences, West Virginia University, Morgantown, WV 26506, USA
[††]United States Department of Agriculture–Agricultural Research Service (USDA-ARS), Arid-Land Agricultural Research Center, Maricopa, AZ 85138, USA

[1]Present address: School of Plant Sciences, University of Arizona, Tucson, AZ 85721, USA

<u>Corresponding author:</u>
Michael A. Gore
358 Plant Science Building, Cornell University, Ithaca, NY 14853
Tel: +1 (607) 255-5492
E-mail: mag87@cornell.edu

File S1-S4

File S1. Best linear unbiased predictors (BLUPs) for soil elemental concentrations within the experimental field site. The Microsoft Excel file contains the BLUPs from the mixed linear model fitted for the soil samples taken at five depths from each of the neutron probe access sites in years 2010 and 2012. The Universal Transverse Mercator (UTM, based on North American Datum 1983) coordinates, X UTM and Y UTM positions, are provided for each of the neutron probe access sites. Elemental concentrations are reported in parts per million (ppm).

File S2. Best linear unbiased estimators (BLUEs) for seed elemental concentrations. The Microsoft Excel file contains the BLUEs from the fitted mixed linear model for both the overall and by-year BLUEs calculated for the 95 recombinant inbred lines (RILs) and parents of the TM-1×NM24016 mapping population evaluated under water-limited (WL) and well-watered (WW) conditions. Parental lines were excluded for the purposes of quantitative trait loci (QTL) mapping. Seed elemental concentrations are reported as parts per billion (ppb).

File S3. Marker genotype data for the TM-1×NM24016 mapping population. The Microsoft Excel file contains the marker genotype scores for the 95 recombinant inbred lines (RILs) from the TM-1×NM24016 mapping population that were used in this work. The first three columns provide the genetic linkage map information from Gore, M. A. et al. 2014. *The Plant Genome* 7:1-10. Genotype marker data for the RILs are reported in columns D through CT, with a header labeled as NMXX where XX denotes the unique numeric identifier for each RIL that corresponds with the BLUEs in File S2. Marker data are coded for ICIM v. 4.0 with 2, 1, and 0 designating the homozygous parent 1 (TM-1), heterozygote, and homozygous parent 2 (NM24016) genotypic states, respectively, and -1 representing missing genotypic data. Genetic distances are reported in centiMorgans (cM).

File S4. Integration of the TM-1×NM24016 genetic linkage map with the *G. hirsutum* L. acc. TM-1 draft genome sequence. The Microsoft Excel file contains the genetic linkage map information from Gore, M. A. et al. 2014. *The Plant Genome* 7:1-10, and the results from aligning marker context sequences to the *G. hirsutum* L. acc. TM-1 draft genome sequence (NBI assembly v1.1, Zhang, T. et al. 2015. *Nature Biotechnology* 33:531-537). A complete description of the alignment process, including software used, is described in Pauli, D. et al. 2016a. *G3* 6:865-879. The assignment of linkage groups to the draft genome sequence do not represent definitive placement of markers with respect to physical position on the TM-1 genome.

Supplemental Table 1. F-values for fixed effects from the analysis of variance (ANOVA) of soil samples collected from the experimental field in which the TM1×NM24016 recombinant inbred line (RIL) population was evaluated at the Maricopa Agricultural Center in Maricopa, AZ. Soil sampling was performed in 2010 and 2012, with samples being collected at 5 depths: 0 – 30; 30 – 60; 60 – 90; 90 – 120; and 120 – 150 cm.

Element	Year	Depth
As	0.52^{NS}	42.80****
Ca	7.64*	134.70****
Co	14.64*	63.16****
Cu	9.78*	207.10****
Fe	11.43*	43.17****
K	84.95***	120.80****
Mg	7.75*	3.80**
Mn	11.91*	176.50****
Mo	0.41 ^{NS}	6.49****
Ni	18.70*	70.06****
P	0.22 ^{NS}	39.62****
Rb	30.10**	109.90****
S	82.15****	26.86****
Zn	31.01**	92.20****

NS Not Significant at the < 0.05 level.
* Significant at the < 0.05 level.
** Significant at the < 0.01 level.
*** Significant at the < 0.001 level.
**** Significant at the < 0.0001 level.

Supplemental Table 2. Geostatistical model parameters used for interpolation of soil element concentrations (kriging) across the experimental field at the Maricopa Agricultural Center in Maricopa, AZ, using the estimated best linear unbiased predictors (BLUPs) from a fitted linear mixed model. “Variance model” is the covariance structure used to account for spatial relationships of sampled data points. “Nugget” is the measurement error plus the variation that occurs over distances less than the shortest sampling interval. “Range” is the distance (m) at which spatial dependencies are no longer present, i.e., sampling points are spatially independent, and sill is the maximum variance at which this point occurs. SS Error, sums of squares error for the fitted model; NA, not applicable.

Element	Log transformation	Variance model	Nugget	Range (m)	Sill	SS Error
As	No	Spherical	9.40E-02	16.79	4.42E-01	7.29E-03
Ca	Yes	Spherical	1.47E-02	15.35	5.31E-02	1.97E-05
Co	No	Gaussian	2.91E-02	20.30	2.99E-02	7.94E-05
Cu	No	Spherical	1.91E-01	24.34	5.19E-01	9.90E-03
Fe	Yes	Pentaspherical	5.67E-04	31.61	4.46E-04	1.23E-08
K	Yes	Bessel	5.67E-04	12.49	1.19E-04	2.14E-09
Mg	Yes	Pentaspehrical	2.36E-03	24.98	1.23E-02	4.08E-06
Mn	No	Pentaspehrical	2.26E+01	66.44	3.04E+01	3.01E+01
Mo	No	Pentaspherical	1.08E-03	12.59	3.91E-04	7.12E-08
Ni	No	Spherical	5.71E-02	26.51	1.14E-01	3.51E-04
P	Yes	Spherical	3.97E-03	20.69	1.07E-02	2.12E-06
Rb	No	Pentaspherical	6.35E-01	39.09	7.09E-01	5.47E-02
S	Yes	Nugget	2.60E-04	NA	1.88E-12	7.44E-09
Zn	No	Pentaspherical	1.51E+00	35.72	1.33E+00	2.54E-01

Supplemental Table 3. Summary statistics for interpolated soil element concentrations. Means, standard deviations, minimum, maximum, and range of soil element concentrations (in parts per million) interpolated across the experimental field at the Maricopa Agricultural Center in Maricopa, AZ. Soil samples were collected from five depths at neutron probe installation sites that were distributed throughout the field in 2010 and 2012. For sulfur, no spatial pattern was observed. Therefore, only the nugget variance was used for modeling purposes, resulting in one interpolated value for the entire experimental field area. Nugget variance represents the measurement error plus the variation that occurs over distances less than the shortest sampling interval. NA, not available.

Element	Mean	Std. Dev.	Min.	Max.	Range
As	3.72	0.41	2.73	4.99	2.26
Ca	8784.91	1092.17	5929.91	12809.64	6879.73
Co	4.50	0.11	4.18	4.75	0.57
Cu	15.42	0.49	13.95	16.51	2.57
Fe	9184.29	122.20	8775.33	9424.81	649.48
K	10140.95	104.14	9862.72	10353.72	491.00
Mg	4774.15	311.36	3778.03	5455.11	1677.09
Mn	256.44	3.88	244.98	264.99	20.02
Mo	0.70	0.01	0.68	0.73	0.04
Ni	7.95	0.23	7.15	8.53	1.37
P	441.16	27.01	365.17	508.53	143.36
Rb	21.76	0.58	19.70	23.32	3.62
S	1089.90	NA	NA	NA	NA
Zn	41.13	0.73	38.69	42.99	4.30

Supplemental Table 4. Fixed effects for elements. F-values for fixed effects from an analysis of variance (ANOVA) for the TM-1×NM24016 recombinant inbred line (RIL) population, its two parents, and commercial check varieties for elements analyzed in seed. Covariate indicates whether flowering time (i.e., time to first flower) or soil element concentration had a significant linear relationship with seed element concentration. Data were collected from 2010-12 at the Maricopa Agricultural Center located in Maricopa, AZ.

Ion	Covariate type	Covariate	Genotype	Irrigation regime	Genotype*Irrigation regime
As	Flowering	4.69*	4.81****	2.30 ^{NS}	1.00 ^{NS}
Ca			5.00****	6.97*	0.85 ^{NS}
Co	Soil element	13.70***	8.98****	0.11 ^{NS}	1.66***
Cu	Flowering	4.26*	18.86****	27.54***	1.26 ^{NS}
Fe			8.83****	12.68**	0.93 ^{NS}
K			5.11****	1.26 ^{NS}	1.04 ^{NS}
Mg	Soil element	6.83**	9.82****	30.73***	1.20 ^{NS}
Mn			11.75****	1.80 ^{NS}	1.53**
Mo			3.24****	40.67***	1.30*
Ni	Flowering	8.80**	10.76****	2.14 ^{NS}	1.04 ^{NS}
P			6.29****	3.17 ^{NS}	0.67 ^{NS}
Rb	Soil element	46.89****	5.17****	4.07 ^{NS}	1.01 ^{NS}
S			8.34****	12.02**	8.34****
Zn			9.06****	19.99**	1.23 ^{NS}

^{NS} Not Significant at the < 0.05 level.
* Significant at the < 0.05 level.
** Significant at the < 0.01 level.
*** Significant at the < 0.001 level.
**** Significant at the < 0.0001 level.

Supplemental Table 5. Phenotypic ($\hat{r}_{pij}$) correlations with standard errors in parenthesis between the 14 elements evaluated in the TM-1×NM24016 recombinant inbred line (RIL) mapping population evaluated under contrasting irrigation regimes, water-limited (WL, values above the diagonal) and well-watered (WW, below the diagonal). Field trials were conducted from 2010-12 at the Maricopa Agricultural Center located in Maricopa, AZ.

	Mg	Cu	Ca	Ni	Co	As	Rb	Fe	K	Mn	Mo	P	S	Zn
Mg		0.35 (0.09)	0.45 (0.08)	0.36 (0.09)	0.11 (0.10)	-0.35 (0.11)	0.22 (0.10)	0.54 (0.07)	0.01 (0.10)	0.55 (0.07)	0.14 (0.10)	0.66 (0.06)	0.22 (0.09)	0.66 (0.06)
Cu	0.39 (0.08)		0.30 (0.09)	0.19 (0.10)	0.15 (0.10)	0.06 (0.10)	0.19 (0.10)	0.47 (0.08)	0.04 (0.10)	0.34 (0.09)	0.24 (0.09)	0.35 (0.09)	0.24 (0.09)	0.46 (0.08)
Ca	0.43 (0.08)	0.20 (0.10)		0.32 (0.09)	0.13 (0.10)	-0.26 (0.11)	-0.03 (0.10)	0.35 (0.09)	-0.13 (0.10)	0.60 (0.06)	0.11 (0.10)	0.48 (0.08)	0.09 (0.10)	0.43 (0.08)
Ni	0.41 (0.08)	0.25 (0.09)	0.33 (0.09)		0.31 (0.09)	-0.33 (0.13)	0.29 (0.09)	0.50 (0.07)	0.00 (0.10)	0.39 (0.08)	0.18 (0.09)	0.50 (0.07)	-0.03 (0.10)	0.56 (0.07)
Co	0.14 (0.10)	0.25 (0.09)	0.13 (0.10)	0.35 (0.09)		0.13 (0.10)	0.06 (0.10)	0.30 (0.09)	0.28 (0.09)	0.30 (0.09)	0.22 (0.09)	0.22 (0.09)	0.06 (0.10)	0.36 (0.09)
As	-0.29 (0.11)	-0.03 (0.10)	-0.32 (0.11)	-0.26 (0.12)	0.07 (0.10)		-0.04 (0.11)	-0.24 (0.12)	0.17 (0.09)	-0.07 (0.11)	0.01 (0.10)	-0.26 (0.12)	0.08 (0.10)	-0.31 (0.13)
Rb	0.20 (0.10)	0.16 (0.10)	0.17 (0.10)	0.24 (0.09)	0.08 (0.10)	-0.09 (0.11)		0.19 (0.09)	0.33 (0.09)	0.25 (0.09)	0.07 (0.10)	0.05 (0.10)	0.03 (0.10)	0.28 (0.09)
Fe	0.53 (0.07)	0.54 (0.07)	0.32 (0.09)	0.60 (0.06)	0.39 (0.08)	-0.15 (0.11)	0.14 (0.10)		0.22 (0.09)	0.59 (0.06)	0.17 (0.09)	0.47 (0.08)	0.16 (0.10)	0.77 (0.04)
K	-0.10 (0.10)	0.05 (0.10)	-0.11 (0.10)	-0.03 (0.10)	0.10 (0.10)	0.02 (0.10)	0.30 (0.09)	0.07 (0.10)		0.30 (0.09)	0.01 (0.10)	-0.04 (0.10)	0.12 (0.10)	0.01 (0.10)
Mn	0.59 (0.06)	0.37 (0.09)	0.51 (0.07)	0.44 (0.08)	0.30 (0.09)	-0.19 (0.12)	0.28 (0.09)	0.57 (0.07)	0.15 (0.10)		0.18 (0.09)	0.36 (0.09)	0.08 (0.10)	0.57 (0.07)
Mo	0.13 (0.10)	0.27 (0.09)	0.19 (0.09)	0.33 (0.09)	0.23 (0.09)	0.00 (0.10)	0.01 (0.10)	0.29 (0.09)	0.02 (0.10)	0.21 (0.09)		0.21 (0.09)	0.40 (0.08)	0.23 (0.09)
P	0.63 (0.06)	0.43 (0.08)	0.43 (0.08)	0.57 (0.07)	0.23 (0.09)	-0.20 (0.12)	0.11 (0.10)	0.52 (0.07)	-0.05 (0.10)	0.44 (0.08)	0.34 (0.08)		0.16 (0.10)	0.65 (0.06)
S	0.24 (0.09)	0.18 (0.10)	0.15 (0.10)	0.19 (0.10)	0.13 (0.10)	0.04 (0.10)	0.11 (0.10)	0.20 (0.09)	0.22 (0.09)	0.16 (0.10)	0.52 (0.07)	0.29 (0.09)		0.14 (0.10)
Zn	0.64 (0.06)	0.51 (0.07)	0.41 (0.08)	0.61 (0.06)	0.35 (0.09)	-0.30 (0.13)	0.23 (0.09)	0.72 (0.05)	-0.06 (0.10)	0.55 (0.07)	0.28 (0.09)	0.64 (0.06)	0.27 (0.09)	

Supplemental Table 6. Genotypic ($\hat{r}_{gij}$) correlations with standard errors in parenthesis between the 14 elements evaluated in the TM-1×NM24016 recombinant inbred line (RIL) mapping population evaluated under contrasting irrigation regimes, water-limited (WL, values above the diagonal) and well-watered (WW, below the diagonal). Field trials were conducted from 2010-12 at the Maricopa Agricultural Center located in Maricopa, AZ.

	Mg	Cu	Ca	Ni	Co	As	Rb	Fe	K	Mn	Mo	P	S	Zn
Mg		0.36 (0.09)	0.46 (0.08)	0.37 (0.09)	0.11 (0.10)	-0.35 (0.11)	0.22 (0.10)	0.55 (0.07)	0.01 (0.10)	0.57 (0.07)	0.14 (0.10)	0.67 (0.06)	0.22 (0.10)	0.67 (0.06)
Cu	0.40 (0.09)		0.30 (0.09)	0.19 (0.10)	0.15 (0.10)	0.06 (0.10)	0.19 (0.10)	0.48 (0.08)	0.04 (0.10)	0.35 (0.09)	0.25 (0.10)	0.37 (0.09)	0.25 (0.10)	0.46 (0.08)
Ca	0.44 (0.08)	0.20 (0.10)		0.33 (0.09)	0.13 (0.10)	-0.26 (0.11)	-0.03 (0.10)	0.36 (0.09)	-0.13 (0.10)	0.61 (0.07)	0.11 (0.10)	0.50 (0.08)	0.09 (0.10)	0.44 (0.08)
Ni	0.41 (0.09)	0.25 (0.10)	0.34 (0.09)		0.31 (0.09)	-0.34 (0.13)	0.29 (0.09)	0.52 (0.08)	0.00 (0.10)	0.40 (0.09)	0.19 (0.10)	0.51 (0.08)	-0.04 (0.10)	0.56 (0.07)
Co	0.14 (0.10)	0.26 (0.10)	0.13 (0.10)	0.36 (0.09)		0.13 (0.10)	0.06 (0.10)	0.31 (0.09)	0.28 (0.10)	0.31 (0.09)	0.21 (0.10)	0.24 (0.10)	0.07 (0.10)	0.37 (0.09)
As	-0.30 (0.11)	-0.03 (0.10)	-0.32 (0.11)	-0.27 (0.13)	0.07 (0.10)		-0.04 (0.11)	-0.24 (0.13)	0.17 (0.10)	-0.07 (0.11)	0.01 (0.11)	-0.26 (0.13)	0.09 (0.10)	-0.32 (0.13)
Rb	0.21 (0.10)	0.16 (0.10)	0.17 (0.10)	0.25 (0.09)	0.09 (0.10)	-0.09 (0.11)		0.20 (0.09)	0.33 (0.09)	0.26 (0.09)	0.07 (0.10)	0.05 (0.10)	0.03 (0.10)	0.28 (0.09)
Fe	0.55 (0.07)	0.55 (0.07)	0.33 (0.09)	0.62 (0.07)	0.41 (0.09)	-0.16 (0.12)	0.15 (0.10)		0.23 (0.10)	0.61 (0.07)	0.17 (0.10)	0.48 (0.08)	0.16 (0.10)	0.78 (0.04)
K	-0.10 (0.10)	0.05 (0.10)	-0.11 (0.10)	-0.03 (0.10)	0.09 (0.10)	0.02 (0.11)	0.31 (0.09)	0.06 (0.1)		0.30 (0.09)	0.00 (0.10)	-0.05 (0.10)	0.12 (0.10)	0.01 (0.10)
Mn	0.61 (0.07)	0.37 (0.09)	0.51 (0.08)	0.45 (0.08)	0.30 (0.09)	-0.19 (0.12)	0.28 (0.09)	0.59 (0.07)	0.15 (0.10)		0.18 (0.10)	0.37 (0.09)	0.08 (0.10)	0.57 (0.07)
Mo	0.14 (0.10)	0.28 (0.10)	0.19 (0.10)	0.36 (0.09)	0.24 (0.10)	0.00 (0.11)	0.01 (0.11)	0.30 (0.1)	0.01 (0.10)	0.21 (0.10)		0.23 (0.10)	0.41 (0.09)	0.24 (0.10)
P	0.64 (0.06)	0.44 (0.08)	0.44 (0.08)	0.59 (0.07)	0.24 (0.10)	-0.21 (0.12)	0.11 (0.10)	0.52 (0.08)	-0.07 (0.10)	0.45 (0.08)	0.37 (0.09)		0.17 (0.10)	0.66 (0.06)
S	0.24 (0.10)	0.18 (0.10)	0.15 (0.10)	0.20 (0.10)	0.13 (0.10)	0.04 (0.01)	0.11 (0.10)	0.20 (0.10)	0.22 (0.10)	0.17 (0.10)	0.54 (0.08)	0.29 (0.10)		0.14 (0.10)
Zn	0.65 (0.06)	0.51 (0.08)	0.41 (0.09)	0.61 (0.06)	0.36 (0.09)	-0.31 (0.13)	0.24 (0.09)	0.73 (0.05)	-0.06 (0.10)	0.55 (0.07)	0.29 (0.10)	0.66 (0.06)	0.28 (0.10)	

Supplemental Table 7. Summary of quantitative trait loci (QTL) mapped using Bayesian classification mapping for 14 elements in the TM-1×NM24016 recombinant inbred line (RIL) population evaluated under contrasting irrigation regimes, water-limited (WL) and well-watered (WW) conditions, at the Maricopa Agricultural Center, Maricopa, AZ. Quantitative trait loci (QTL) significance is based on the reported Bayes factor with values larger than 100 indicating "decisive evidence" for a QTL, see Jeffreys (1961) for further details. Marker positions are reported as centimorgans (cM).

Element	QTL name	Irrigation regime[a]	Chr.[b]	LG[c]	Peak marker position (cM)	Peak marker	Bayes factor[d]	Beta[e]	SD[f]
As	*qAs.A12.45.18*	WW	A12	45	18.12	SNP0327	884.70	0.45	0.13
	qAs.A05.75.09	WW	A05	75	9.29	SNP0099	263.91	0.41	0.21
	qAs.D06.110.12	WW	D06	110	12.38	DPL0080a	1.95E+07	0.68	0.21
	qAs.D06.112.04	WW	D06	112	3.67	BNL1047a	1.76E+06	0.63	0.26
Ca	*qCa.A06.19.07*	WL	A06	19	7.31	SNP0479	800.14	-0.54	0.20
	qCa.A12.45.18	WW	A12	45	18.12	SNP0327	214.06	-0.48	0.18
	qCa.A06.117.22	WW	A06	117	21.66	SNP0031	145.83	-0.46	0.23
Cu	*qCu.A07.28.00*	WL	A07	28	0.00	DC30012a	4.50E+15	-1.38	0.31
	qCu.A07.28.00	WW	A07	28	0.00	DC30012a	1484.98	-0.54	0.28
	qCu.D01.64.20	WW	D01	64	19.55	SNP0026	134.95	-0.46	0.18
Fe	*qFe.A05.74.16*	WL	A05	74	16.41	SNP0316	2.87E+06	-0.71	0.21
	qFe.D12.116.00	WL	D12	116	0.00	C2-055a	240.34	-0.44	0.16
K	*qK.A09.33.00*	WW	A09	33	0.00	SHIN-0817a	1.41E+04	0.63	0.15
	qK.A10.87.03	WW	A10	87	3.31	SNP0312	1128.73	0.52	0.23
	qK.A11.91.25	WW	A11	91	24.87	SNP0382	1.26E+09	0.88	0.15
Mg	*qMg.A11.44.27*	WL	A11	44	27.39	NAU2152a	1511.42	-0.49	0.15
	qMg.A12.45.21	WL	A12	45	21.39	SNP0047	1.37E+04	-0.53	0.14
	qMg.A12.45.21	WW	A12	45	21.39	SNP0047	898.58	-0.52	0.16
	qMg.D07.66.21	WL	D07	66	21.30	BNL1227a	117.73	-0.39	0.15
	qMg.A05.74.16	WL	A05	74	16.41	SNP0316	194.18	-0.40	0.21
	qMg.A06.117.22	WW	A06	117	21.66	SNP0031	1329.09	-0.53	0.19
Mo	*qMo.A02.69.00*	WL	A02	69	0.00	JESPR101a	283.57	-0.49	0.16
Ni	*qNi.D01.64.20*	WL	D01	64	19.55	SNP0026	120.92	-0.38	0.17

	qNi.A05.74.05	WW	A05	74	4.77	SNP0055	795.22	-0.49	0.15
	qNi.A05.74.13	WL	A05	74	12.52	SNP0035	4176.56	-0.46	0.21
	qNi.D12.116.00	WL	D12	116	0.00	C2-055a	155.76	-0.38	0.14
	qNi.D12.116.00	WW	D12	116	0.00	C2-055a	582.49	-0.47	0.24
P	*qP.A03.06.34*	WL	A03	6	33.92	SNP0130	7960.08	-0.62	0.15
	qP.A11.43.14	WL	A11	43	14.42	DPL1121a	170.76	-0.47	0.18
	qP.A05.74.05	WL	A05	74	4.77	SNP0055	102.23	-0.45	0.17
	qP.A05.74.05	WW	A05	74	4.77	SNP0055	3.12E+05	-0.70	0.24
Rb	*qRb.A05.14.02*	WW	A05	14	2.02	SNP0155	1492.16	-0.51	0.16
	qRb.A08.30.14	WW	A08	30	13.63	SNP0214	392.68	-0.45	0.23
	qRb.A12.45.00	WW	A12	45	0.00	SNP0264	115.62	-0.42	0.19
	qRb.D10.83.18	WW	D10	83	17.96	SHIN-1586a	7471.98	-0.56	0.15
	qRb.D06.112.25	WW	D06	112	25.00	DC30135a	247.30	-0.45	0.15
Zn	*qZn.A05.74.13*	WW	A05	74	12.52	SNP0035	1050.32	-0.52	0.22
	qZn.A05.74.16	WL	A05	74	16.41	SNP0316	133.68	-0.43	0.27

a. Irrigation regime, irrigation regime for which a QTL was identified.
b. Chr., chromosome on which the marker is located based on Pauli et al. (2016a).
c. LG, linkage group.
d. Bayes factor, converted posterior probability indicating likelihood of QTL presence.
e. Beta, fitted regression coefficient for respective marker in model.
f. SD, standard deviation of the regression coefficient, beta, from fitted model.

Supplemental Table 8. Summary of significant quantitative trait loci (QTL) identified using the multi-trait analysis approach of seemingly unrelated regression (SUR) to map two independent groupings of elements in the TM-1×NM24016 recombinant inbred line (RIL) population evaluated under contrasting irrigation regimes, water-limited (WL) and well-watered (WW) conditions, at the Maricopa Agricultural Center, Maricopa, AZ. Select elements were combined into biologically relevant groupings based on biochemical function as outlined in Taiz and Zeiger (2006) and Mengel and Kirkby (2012). The “ionic” group consists of calcium, potassium, magnesium, and manganese, whereas the “redox” group is composed of iron, zinc, copper, nickel, and molybdenum. The *P*-values associated with the respective marker is significant at a Bonferroni-corrected threshold of α = 0.05. Marker positions are reported as centimorgans (cM).

Category	QTL name	Peak Marker	Chr.[a]	LG[b]	Peak Marker Position (cM)	Irrigation regime	SUR *P*-value
Redox	*qRedox.A03.05.14*	SNP0023	A03	5	13.94	WW	9.96E-08
Redox	*qRedox.A03.06.27*	SNP0286	A03	6	26.83	WL	2.44E-08
						WW	6.58E-08
Ionic	*qIonic.A03.09.10*	SHIN-0473a	A03	9	10.34	WL	5.60E-06
Redox	*qRedox.A06.17.14*	SNP0191	A06	17	14.08	WW	1.34E-05
Ionic	*qIonic.A06.19.07*	SNP0479	A06	19	7.31	WL	5.08E-05
Redox	*qRedox.A06.19.11*	SNP0404	A06	19	10.62	WL	3.78E-05
Ionic	*qIonic.A07.24.17*	CIR238a	A07	24	16.60	WW	4.94E-05
Redox	*qRedox.A07.28.00*	DC30012a	A07	28	0.00	WL	1.36E-09
						WW	3.88E-05
Ionic	*qIonic.A09.33.17*	TMB2483a	A09	33	17.12	WL	1.18E-06
						WW	9.14E-06
Ionic	*qIonic.A11.39.01*	DPL1931a	A11	39	0.53	WL	8.46E-06
						WW	4.47E-05
Ionic	*qIonic.A11.41.02*	DPL1379a	A11	41	1.57	WL	1.10E-06
						WW	1.49E-06
Ionic	*qIonic.A11.42.06*	SNP0258	A11	42	5.86	WL	1.54E-05
Redox	*qRedox.A11.44.00*	SNP0058	A11	44	0.00	WW	4.28E-05
Ionic	*qIonic.A12.47.06*	SNP0265	A12	47	6.11	WL	5.59E-05
Ionic	*qIonic.A12.50.01*	BNL0666	A12	50	0.69	WL	2.31E-05
Redox	*qRedox.D12.52.07*	SNP0348	D12	52	6.58	WL	1.41E-05

						WW	3.19E-06
Ionic	*qIonic.D02.59.00*	BNL4061a	D02	59	0.00	WL	2.02E-05
						WW	1.96E-05
Ionic	*qIonic.A01.61.09*	TMB1931a	A01	61	9.32	WL	5.67E-05
Redox	*qRedox.D01.64.20*	SNP0026	D01	64	19.55	WL	9.02E-07
						WW	1.10E-06
Redox	*qRedox.A02.69.00*	JESPR101a	A02	69	0.00	WL	4.58E-06
Redox	*qRedox.A05.74.05*	SNP0055	A05	74	4.77	WL	6.84E-09
						WW	5.66E-08
Redox	*qRedox.A05.74.16*	SNP0316	A05	74	16.41	WL	6.24E-09
						WW	5.62E-07
Ionic	*qIonic.D05.76.13*	DPL0595a	D05	76	12.82	WL	7.85E-07
						WW	6.91E-07
Ionic	*qIonic.D05.80.17*	SNP0196	D05	80	16.96	WW	1.28E-05
Redox	*qRedox.D10.88.10*	SNP0064	D10	88	9.85	WL	1.45E-09
						WW	3.48E-06
Ionic	*qIonic.A11.91.23*	SNP0227	A11	91	22.70	WW	2.38E-05
Ionic	*qIonic.D11.92.00*	SHIN-1214b	D11	92	0.00	WL	1.01E-06
						WW	6.61E-06
Ionic	*qIonic.D04.95.00*	BNL4030a	D04	95	0.00	WL	6.06E-06
Ionic	*qIonic.D09.97.05*	SNP0114	D09	97	4.77	WW	3.03E-06
Redox	*qRedox.D08.105.04*	SNP0005	D08	105	3.52	WL	5.28E-05
						WW	2.78E-05
Ionic	*qIonic.A06.117.22*	SNP0031	A06	117	21.66	WW	1.24E-05

a. Chr., chromosome on which the marker is located based on Pauli et al. (2016a).

b. LG, linkage group.

Supplemental Table 9. Comparison of marker loci that were below the critical threshold in the Bayesian classification method (Bayes factor of 100), but were detected using the multi-trait mapping method of seemingly unrelated regression (SUR) in two irrigation regimes, water-limited (WL) and well-watered (WW). Tables A and B display results associated with the multi-element grouping of ionic (Ca, K, Mg, and Mn) and redox (Cu, Fe, Mo, Ni, and Zn) in the multi-trait analysis. The *P*-value associated with the significant marker identified in the multi-trait analysis is significant at a Bonferroni-corrected α = 0.05.

A. Ionic: Ca, K, Mg, and Mn

Marker information				Irrigation Regime[a]	Bayes factors for single elements				SUR *P*-value
Marker	Chr.[b]	LG[c]	Position		Ca	K	Mg	Mn	
TMB2483a	A09	33	17.12	WW	4.48	-	-	-	9.14E-06
DPL1379a	A11	41	1.57	WW	-	-	-	-	1.49E-06
SNP0265	A12	47	6.11	WL	1.02	-	-	-	5.59E-05
BNL0666	A12	50	0.69	WL	-	2.55	-	-	2.31E-05
TMB1931a	A01	61	9.32	WL	2.48	-	-	-	5.67E-05
DPL0595a	D05	76	12.82	WL	-	0.21	-	-	7.85E-07

B. Redox: Cu, Fe, Mo, Ni, and Zn

Marker information				Irrigation Regime[a]	Bayes factors for single elements					SUR *P*-value
Marker	Chr.[b]	LG[c]	Position		Cu	Fe	Mo	Ni	Zn	
SNP0023	A03	5	13.94	WW	-	3.94	-	-	-	9.96E-08
SNP0058	A11	44	0.00	WW	-	-	29.61	-	1.23	4.28E-05
SNP0348	D12	52	6.58	WW	1.54	-	-	-	-	3.19E-06
SNP0005	D08	105	3.52	WW	-	-	-	-	0.61	2.78E-05

a. Irrigation regime, irrigation regime for which a QTL was identified.
b. Chr., chromosome on which the marker is located based on Pauli et al. (2016).
c. LG, linkage group.
d. SUR P-value, P-value from the multi-trait analysis using seemingly unrelated regression.

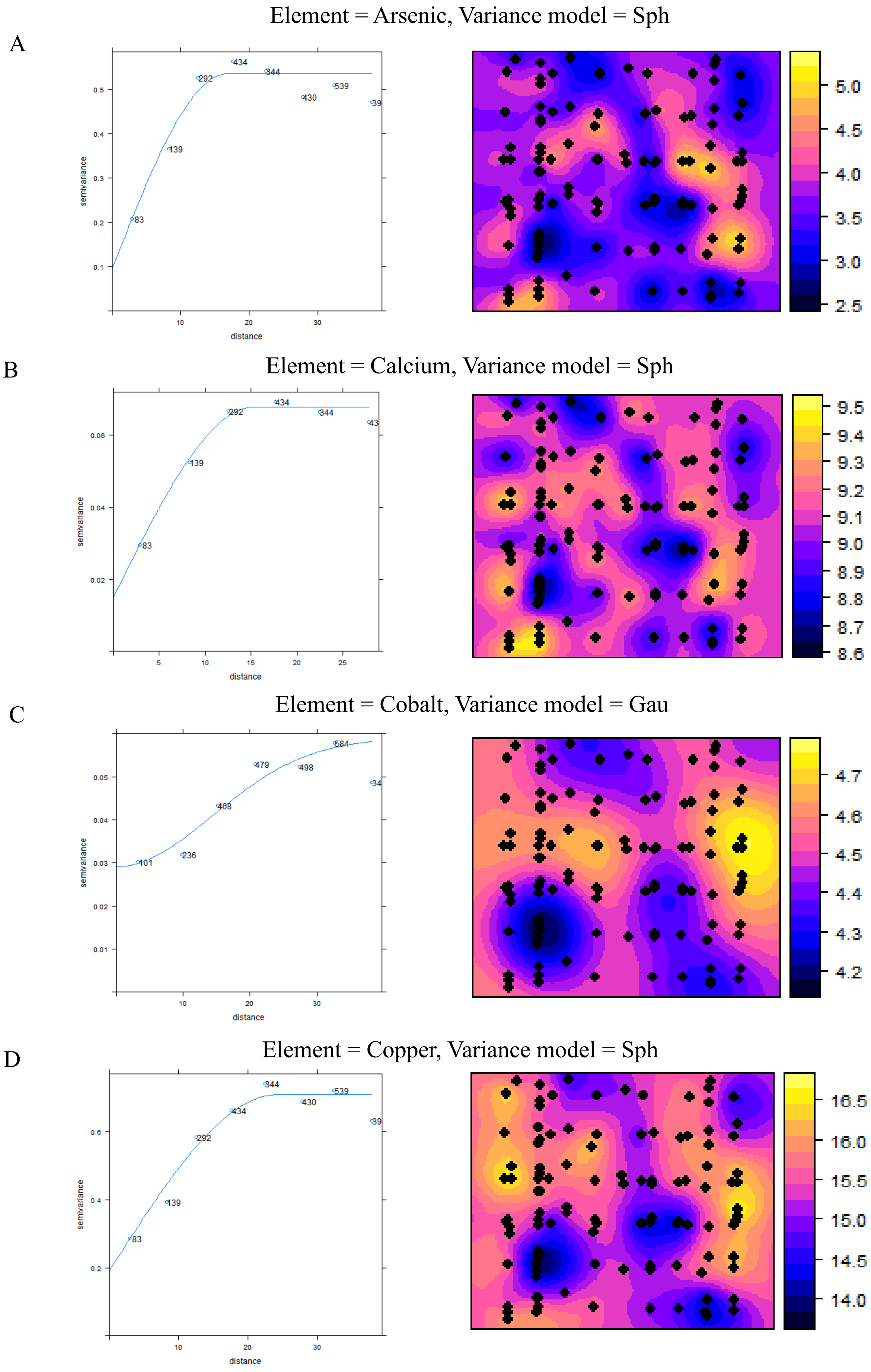

A
Element = Arsenic, Variance model = Sph
semivariance
distance
5.0
4.5
4.0
3.5
3.0
2.5
B
Element = Calcium, Variance model = Sph
semivariance
distance
9.5
9.4
9.3
9.2
9.1
9.0
8.9
8.8
8.7
8.6
C
Element = Cobalt, Variance model = Gau
semivariance
distance
4.7
4.6
4.5
4.4
4.3
4.2
D
Element = Copper, Variance model = Sph
semivariance
distance
16.5
16.0
15.5
15.0
14.5
14.0

E

Element = Iron, Variance model = Pen

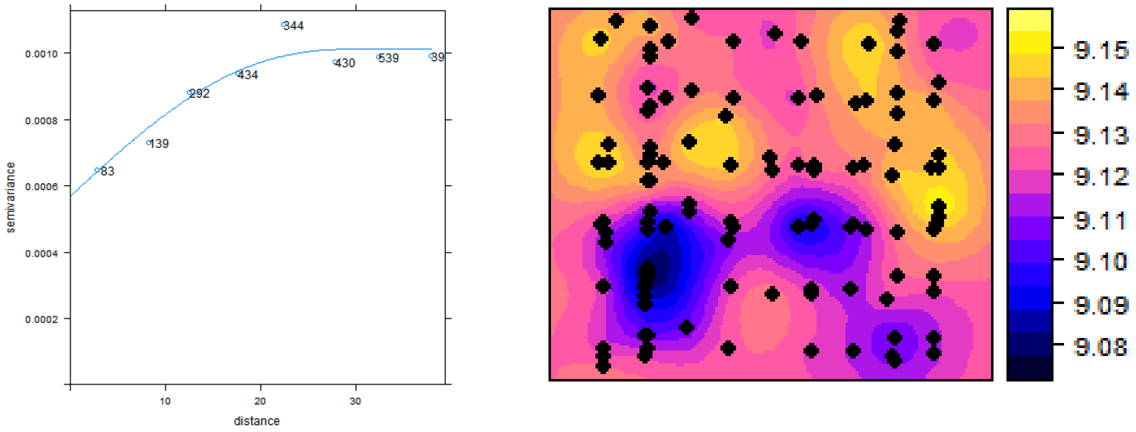

F

Element = Potassium, Variance model = Bes

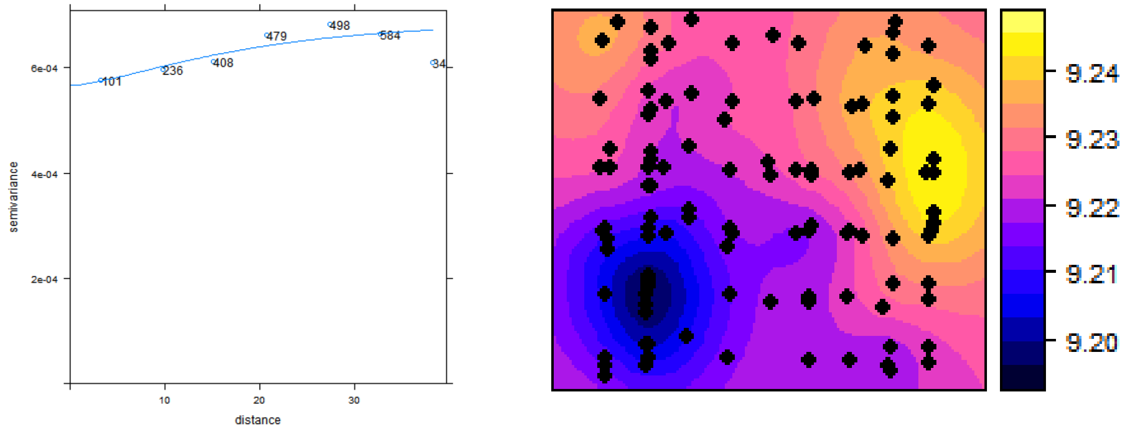

G

Element = Manganese, Variance model = Pen

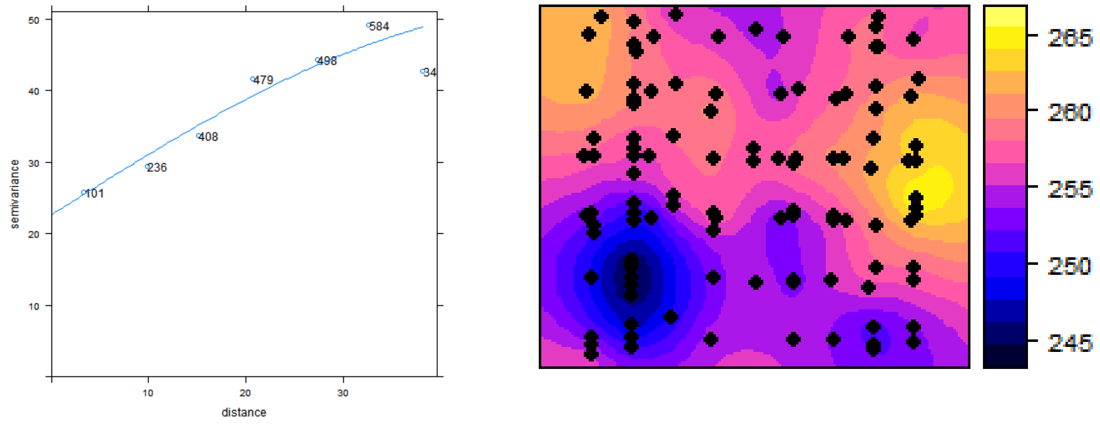

H

Element = Molybdenum, Variance model = Pen

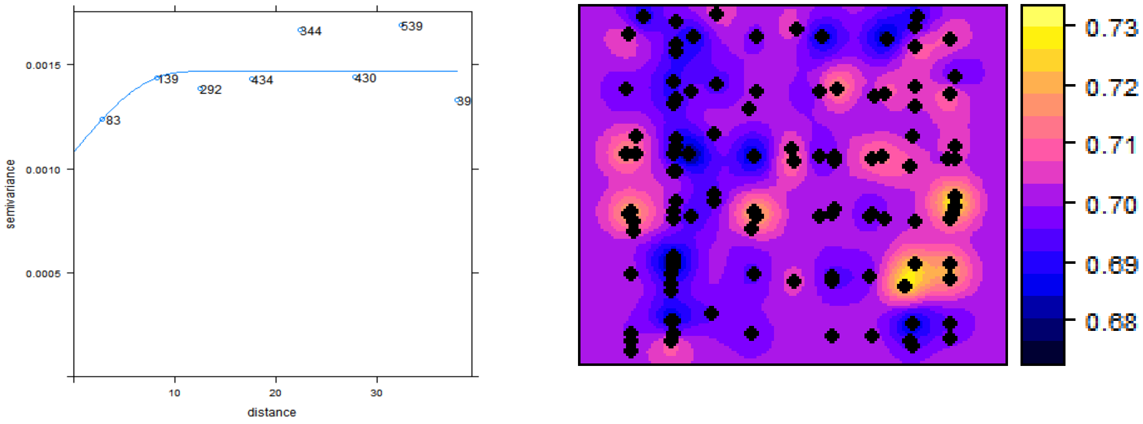

I

Element = Nickel, Variance model = Sph

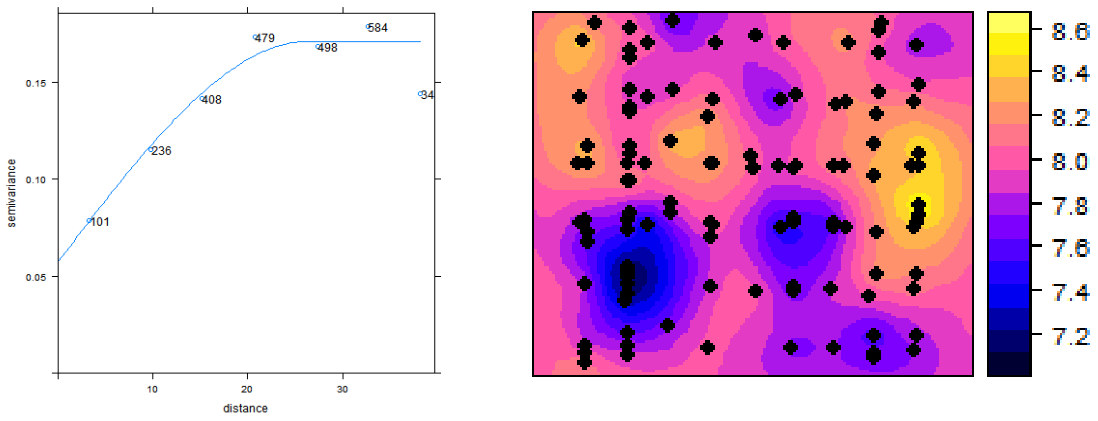

J

Element = Phosphorus, Variance model = Sph

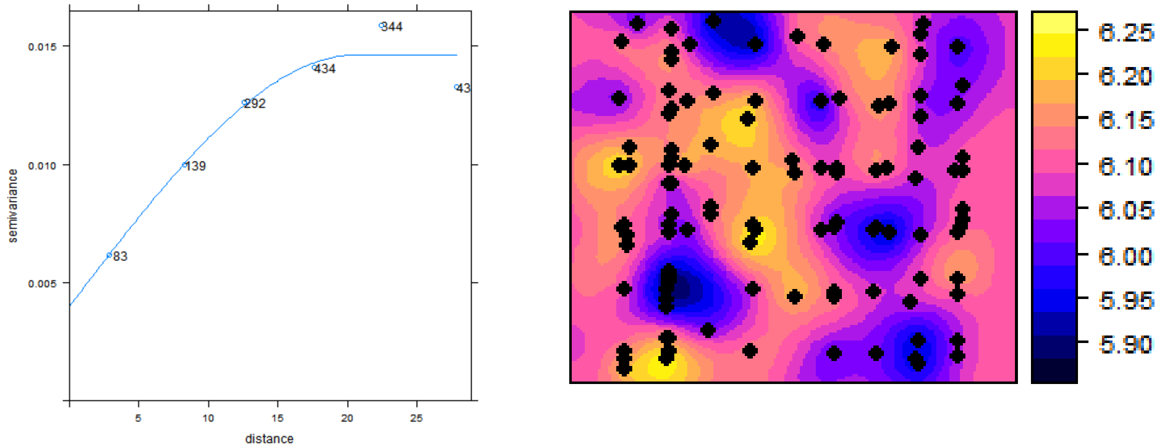

K

Element = Rubidium, Variance model = Pen

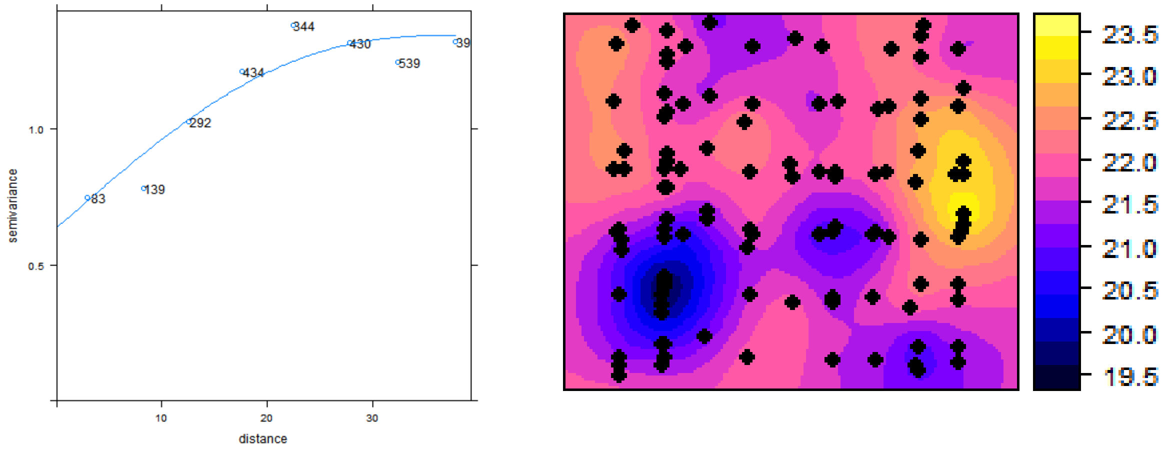

L

Element = Sulfur, Variance model = Nug

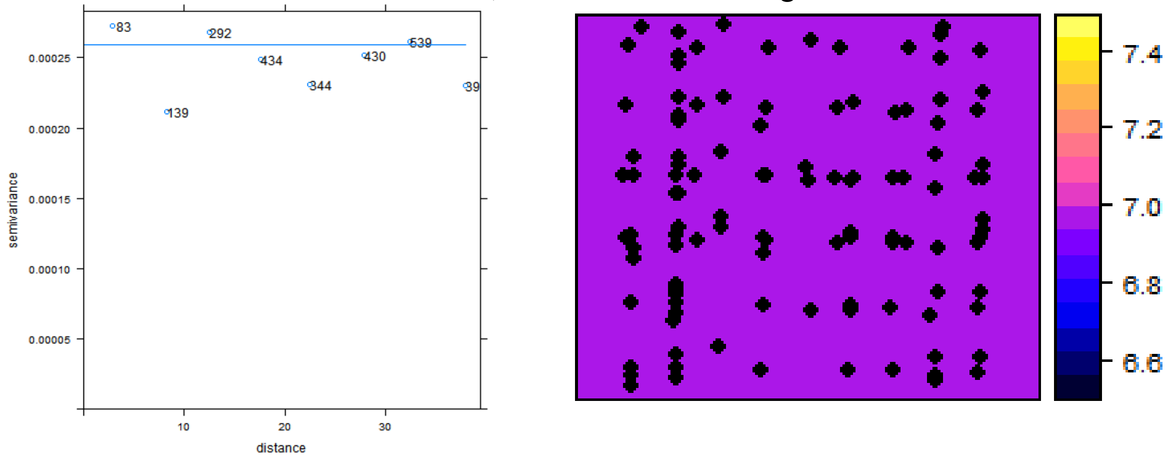

M

Element = Zinc, Variance model = Pen

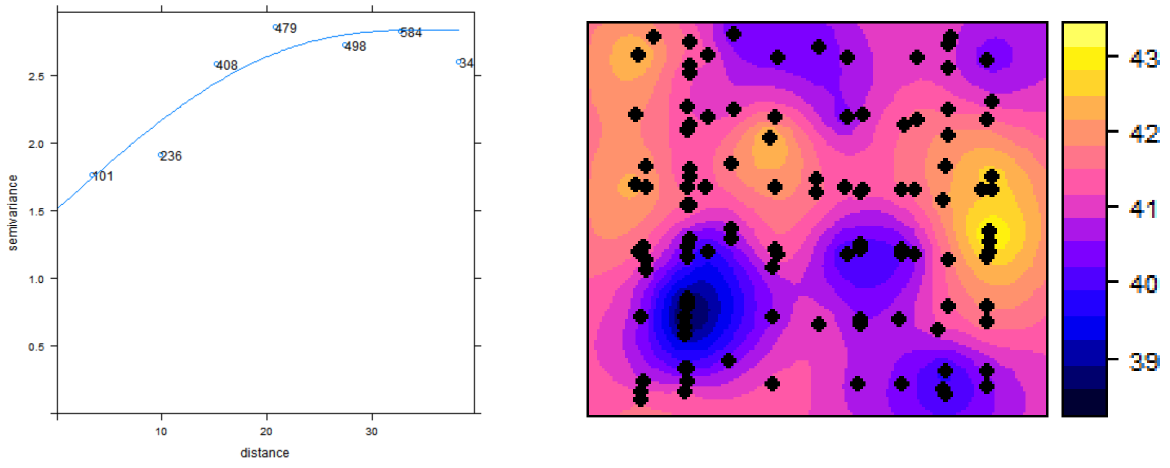

Figure S1. Characterization of soil elemental profile in 2010 and 2012 for A) arsenic, B) calcium, C) cobalt, D) copper, E) iron, F) potassium, G) manganese, H) molybdenum, I) nickel, J) phosphorus, K) rubidium, L) sulfur, and M) zinc in the field site where the mapping population was evaluated. The left-hand panel represents spatial continuity of elemental variation within the field site in terms of the correlation among sampling-site elemental concentrations at a given distance, in meters. Values within the plot area denote the total number of point pairs at the respective distances. The right-hand panel is the interpolated elemental concentrations throughout the field site. The values for arsenic, calcium, iron, phosphorus, and potassium were log transformed prior to model fitting. Variance models used for kriging: Bes, Bessel; Gau, Gaussian; Nug, Nugget, Pen, Pentaspherical; and Sph, spherical.
